# Risk Evaluation of Bogie System Based on Extension Theory and Entropy Weight Method

**DOI:** 10.1155/2014/195752

**Published:** 2014-12-10

**Authors:** Yanping Du, Yuan Zhang, Xiaogang Zhao, Xiaohui Wang

**Affiliations:** ^1^School of Mechanical Engineering, Beijing Institute of Graphic Communication, Beijing 102600, China; ^2^State Key Laboratory of Rail Traffic Control and Safety, Beijing Jiaotong University, Beijing 100044, China; ^3^School of Electromechanical and Vehicle Engineering, Beijing University of Civil Engineering and Architecture, Beijing 100044, China

## Abstract

A bogie system is the key equipment of railway vehicles. Rigorous practical evaluation of bogies is still a challenge. Presently, there is overreliance on part-specific experiments in practice. In the present work, a risk evaluation index system of a bogie system has been established based on the inspection data and experts' evaluation. Then, considering quantitative and qualitative aspects, the risk state of a bogie system has been evaluated using an extension theory and an entropy weight method. Finally, the method has been used to assess the bogie system of four different samples. Results show that this method can assess the risk state of a bogie system exactly.

## 1. Introduction

With the rapid development of China's public rail transport, the train being the most important tool and the direct carrier of passengers' and goods in rail transport, its safety is receiving more and more attention. The safe and efficient operation of a train depends directly on the operation state of its key systems or components. The bogie system directly bears the weight of the carriage and its load and has the role of load bearing, moving, traction, and guiding. It can also mitigate the impact between the wheels and rail, reducing vehicle vibration and ensuring the safe and smooth operation of the train. It can effectively brake, to ensure safe stopping of the train. It is one of the most important parts to support the safe operation of the train. Accurate analysis and evaluation of the operation state of the train bogie system can effectively prevent accidents and improve the ability of active prevention. This can provide support for the train operation management departments in train repair and maintenance, fault prevention, and scheduling decisions.

At present, research on the bogie system of trains has been focused on two aspects. One is the fault diagnosis and state monitoring of the parts involved, for example, the fault diagnosis of wheel sets [1, 2], research on online monitoring systems [3], fault diagnosis [4, 5], and evaluation [6] of bearings, and research on fault diagnosis methods for elastic suspension devices [7–9]. The other approach is a subjective evaluation method that carries out safety evaluation of the bogie system based on expert experience [10].

The above research has had effective results, but on the whole research on the risk analysis and evaluation of the bogie system is still lacking. There may also be overreliance on subjective factors in qualitative assessments. This paper aims to establish a risk evaluation system for the bogie system based on monitored data of the state of key elements of the bogie system and expert experience and uses extension theory and entropy weight method to carry out an evaluation of the bogie system.

## 2. Risk Evaluation System of Bogie Systems

The bogie system consists of the crankcase, wheel set, traction drive device, foundation brake rigging, elastic suspension device, frame, grounding device, and so forth. This paper aims to establish a risk evaluation method for the bogie system using the above mentioned key elements as basic factors.

### 2.1. Evaluation Index for Roller Bearings

At present, fault diagnosis technology for roller bearings based on signal processing of vibration acceleration is mature. It is necessary to accurately detect the status change of roller bearings when its risk state is being evaluated. Therefore, using the vibration signal of roller bearings, calculations can accurately reflect the time domain parameters of the trend variation of roller bearings which can be used as a risk evaluation index. Time domain parameter is a simple method for the detection and diagnosis of early faults of rolling bearings. It includes effective value, peak value, peak factor, kurtosis, pulse factor, margin factor, waveform factor, and so forth [11–14]. Therefore, this paper uses the effective value, peak factor, and kurtosis as risk evaluation indices for bearings. For method of calculation refer to [14].

### 2.2. Evaluation Index for Wheel Sets

Wheel diameter is the diameter of the wheel tread. Friction of wheel treads results in a reduction of wheel diameter, affecting the dynamics of the railcar. The wheel rim is an important part that ensures that the train moves along the track and prevents derailment. A reasonable wheel rim width will ensure the train proceeds safely through the turns and prevents collision of the wheel rim and bolt connections of the rail. As to wheel diameter, to ensure the safety of the train, there are requirements relating to the diameter difference of two wheels in the same shaft and diameter difference of four wheels in the same bogie. Taking the metro vehicle as an example, the range value of wheel diameter is generally 840–770 mm [15–17]. The range value of wheel rim width is 32–26 mm [15–17]. The diameter difference between the left wheel and right wheel in the same shaft should be less than 2 mm [15–17]. The diameter difference between the four wheels in the same bogie should be less than 4 mm [15–17]. Therefore, to evaluate the impact of wheel set risk on the whole bogie system, this paper uses wheel diameter, wheel rim width, difference in diameter between coaxial wheels, and difference in diameter between co-bogie wheels as risk evaluation indices for wheel sets.

### 2.3. Evaluation Index for Traction Drive Devices

Traction motor is often referred to as the “heart” of the train. It is an important part in the normal operation of the train [18]. At present, techniques for fault diagnosis of traction motors can be based on vibration signal, temperature monitoring, electrical current, and so forth [19]. This paper mainly studies the monitoring of hidden risk states of traction motors. It is hoped that the monitoring index can be obtained rapidly, so as to carry out the risk assessment of the bogie system. Therefore, this paper chooses the temperature of the traction motor as the risk evaluation index.

### 2.4. Evaluation Index for Foundation Brake Rigging

Brake shoe of the foundation brake is the most commonly used method in train braking. In the maintenance of metro vehicles, the thickness of the brake shoe must be measured and recorded. Attention is needed when the thickness of the brake shoe is 17.5–18 mm. The brake shoe needs to be changed when its thickness is not more than 15 mm. Brake shoes of different thicknesses have different impact on the operation of the train. It is known from the statistical records of accidents of a local railway network that 56% of braking system faults can be attributed to brake shoe faults.

### 2.5. Other Evaluation Indices

Few specialized sensors are installed on trains for monitoring the state of elastic suspension device, bogie frame, and grounding device. Therefore, for these kinds of devices, risk evaluation is carried out by an expert scoring method. Based on expert experience, we chose risk parameters of the elastic suspension device, bogie frame, and grounding device and fit them into the risk evaluation index system of the bogie system.

### 2.6. Risk Evaluation System for Bogie Systems

**Figure 1:**
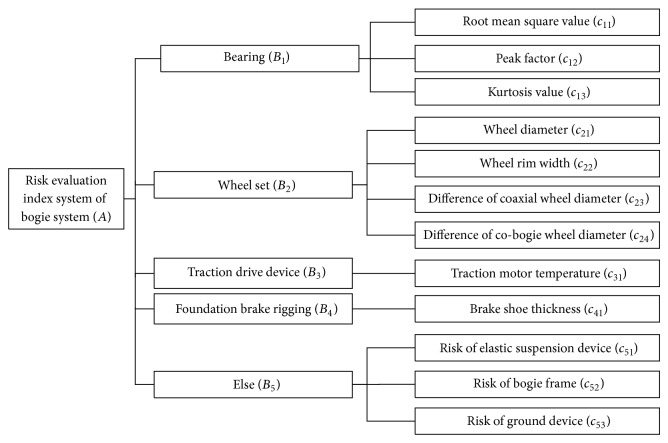
Risk evaluation index system of bogie system.

According to the above analysis and principles, this paper establishes risk evaluation index method for the bogie system, which is shown in the flow chart of Figure 1.

## 3. Extension Theory and Entropy Weight Method

The risk evaluation of the bogie system is a complex and contradictory problem. When carrying out the evaluation, it is necessary to consider quantitative information, such as monitoring and maintenance records, and qualitative knowledge, such as expert experience. Extension theory has been developed in recent years for the study of contradictions in the real world. It mainly deals with contradictory problems from two aspects: the qualitative and the quantitative [20]. The entropy weight method is an objective weighting method [21]. According to the degree of variation of each index, the information entropy can be calculated. The weight of an index is determined by its entropy. The larger the entropy, the smaller the usefulness of the index and, therefore, the smaller the weight. On the contrary, the smaller the entropy, the greater the weight. This paper combines extension theory with the entropy weight method and applies it to the risk evaluation of the bogie system. The steps for risk evaluation based on extension theory and entropy weight method are briefly described below.

*(1) Classification of Risk Level.* If the risk of the evaluation object is classified into *m* levels, the set of comments used in the evaluation is

(1) M=M1,M2,…,M4=hazard level A, hazard level B,…,hazard level m.

*(2) Determination of the Standard Domain and the Extensional Domain.* Standard domain is the range of values of each index corresponding to each hazard level. Standard matter-element can be established based on the evaluation criteria:

(2) R0=MM01M02⋯M0mcv01v02⋯v0n=MM01M02⋯M0mc1a11,b11a12,b12⋯a1m,b1mc2a21,b21a22,b22⋯a2m,b2m⋮⋮⋮⋮cnan1,bn1an2,bn2⋯anm,bnm.

In the formula, *R*_0_ is the same matter-element body of the same matter-elements *R*_1_, *R*_2_,…, *R*_*n*_, and it represents all the status of the evaluation categories; *n* is the number of hazard indexes according to the concept of matter-element theory; *c*_*i*_ is the *i*th risk evaluation index of the object to be evaluated; *v*_0*m*_ is the range of values of *M*_0*m*_ relating to characteristic *c*_*i*_; *M*_0*m*_ is the *m*th evaluation grade of the object to be evaluated; the 〈*a*_*nm*_, *b*_*nm*_〉 that it corresponds to is the standard domain.

Extensional domain is the range of all possible values of an object in its entire life cycle. The extensional domain of the risk level to be evaluated is

(3) RP=MP,c,vP=MPc1vP1c2vP2⋮⋮cnvPn=MPc1aP1,bP1c2aP2,bP2⋮⋮cnaPn,bPn.

In the formula, *M*_*p*_ is all the elements of the comment set; *v*_*pn*_ is the range of values of *c*_*n*_; the 〈*a*_*Pn*_, *b*_*Pn*_〉 that it corresponds to is the extensional domain.

*(3) Determination of the Matter-Element to Be Evaluated.* The matter-element under evaluation refers to the index value of the evaluation object at the time of evaluation:

(4) $$\begin{array}{c} R=\left(M,c,v\right)=\left[\begin{array}{ccc} M & c_{1} & v_{1}\\ & c_{2} & v_{2}\\ & \vdots & \vdots \\ & c_{n} & v_{n} \end{array}\right]. \end{array}$$

In the formula, *R* is the matter-element under evaluation; *M* is the evaluation object; *v*_*i*_ is the value of *c*_*i*_.

*(4) Establishing the Correlation Function to Calculate the Degree of Correlation.* The correlation function of *i*th index of the evaluation object in relation to the level of risk state *j* can be calculated by the following formula:

(5) Kjvi =ρvi,v0jiρvi,vPi−ρvi,v0ji vi∉v0ji−ρvi,v0jiv0ji vi∈v0ji  and  ρvi,vPj=ρvi,v0ji.

In the formula, *K*_*j*_(*v*_*i*_) is the degree of correlation; *ρ*(*v*_*i*_, *v*_0*ji*_), *ρ*(*v*_*i*_, *v*_*Pi*_) are the distances from point *v*_*i*_ to the standard domain and the extensional domain, respectively. Consider

(6) ρvi,v0ji=vi−a0ji+b0ji2−b0ji−a  0ji  2 i=1,2,…,n,ρvi,vPi=vi−aPi+bPi2−bPi−aPi2 i=1,2,…,n.

After the computation, the degree of correlation matrix of each index in relation to hidden risk level *K* = (*k*_*ij*_)_*n*×*m*_ can be obtained.

*(5) Determining the Weights for Each Index.* Then, the entropy weight method is used to calculate the weight of the indices. The detailed process is as follows.

① If the bogie system is classified into *m* levels when evaluated, there will be *n* evaluation indices. Establish a judgment matrix of size *n* × *m*. This is the correlation matrix *K* = (*k*_*ij*_)_*n*×*m*_ solved in the previous section.
② After correlation matrix *s*_*ij*_ = *k*_*ij*_/∑_*j*=1_^*m*^ *k*_*ij*_ is normalized, matrix *S* = (*s*_*ij*_)_*n*×*m*_ can be obtained.
③ Calculate the entropy of the index evaluated using the following formula:
  (7) $$\begin{array}{c} E_{i}=-Q\overset{m}{\underset{j=1}{\sum }}s_{ij}\ln s_{ij}\;\left(i=1,2,3,\ldots ,n;\;j=1,2,3,\ldots ,m\right). \end{array}$$
 If *s*_*ij*_ = 0, then specify *s*_*ij*_ln⁡*s*_*ij*_ = 0; therefore, 0 ≤ *E*_*i*_ ≤ 1:
  (8) $$\begin{array}{c} Q=\frac{1}{\ln n}. \end{array}$$
④ Calculate degree of variation coefficient of the indices using the following formula:
  (9) $$\begin{array}{c} d_{i}=1-E_{i}. \end{array}$$
⑤ Calculate the entropy weight coefficient of the indices using the following formula:
  (10) $$\begin{array}{c} \omega_{i}^{}=\frac{d_{i}}{\sum_{i=1}^{n}d_{i}}. \end{array}$$

*(6) Determination of Risk Level.* Considering the weights of each characteristic, the comprehensive correlative degree is obtained by combining the correlation degree and the weight coefficient:

(11) $$\begin{array}{c} K_{j}\left(M\right)=\overset{n}{\underset{i=1}{\sum }}w_{i}K_{j}\left(v_{i}\right). \end{array}$$

If *K*_*j*0_(*M*) = max⁡{*K*_*j*_(*M*)  *j* = 1,2,…, *m*}, *M* is evaluated as level *j*_0_.

## 4. A Case of Risk Evaluation of Bogie System 

To verify the validity of the method, we chose the bogie system of experimental trains of a metro company as the object of research.

*(1) Classification in Risk Evaluation.* According to the state processing method of bogie systems, the risk level is classified into three grades. The seriousness of risk increases step by step as the risk level proceeds from low to high. Low risk indicates that the bogie system can still function normally; medium risk indicates that the bogie system can function but needs attention; high risk indicates that some fault is present, and corresponding measures should be taken. Therefore, this paper adopts the comments set:

(12) $$\begin{array}{c} M=\left\{M_{1},M_{2},M_{3}\right\}=\left\{\text{low}\;\text{risk},\text{medium}\;\text{risk},\text{high}\;\text{risk}\right\}. \end{array}$$

*(2) Index Criteria.* Data on bearing vibration, wheel set dimension, traction motor temperature, and thickness of brake shoes is collected at the operation field of a metro company. Based on results of data analysis, internal operating procedures, and related trade standards, the index standards are determined, as shown in Table 1.

Select four groups of evaluation sample data as shown in Table 2. 

*(3) Standard Domain and Extensional Domain.* According to Table 1, the standard matter-element and extensional domain matrix are *R*_0_ and *R*_*P*_, respectively. 

*(4) Establishing the Correlation Function to Calculate the Degree of Correlation.* Take sample 1 as an example; according to formulae (5) and (6), the degree of correlation matrix of the three level indexes under indices *B*_1_, *B*_2_, *B*_3_, *B*_4_, and *B*_5_ is *K*_11_, *K*_12_, *K*_13_, *K*_14_, and *K*_15_, respectively:

(13) K11=0.4114−0.5886−0.7120−0.05600.1470−0.25590.2301−0.7699−0.8466,K12=0.1917−0.3485−0.43420.1333−0.8667−0.90000.4000−0.4000−0.55000.1333−0.1333−0.3500,K13=−0.19830.2333−0.0700,K14=−0.4286−0.5714−0.6250,K15=0.4000−0.4000−0.60000.3000−0.7000−0.8000−0.02000.0400−0.3288.

*(5) Calculating the Weight of Indices.* Use the degree of correlation matrix obtained above as input to the entropy weight method.

Use formulae (7)–(10) to calculate the weights of the three level indices:

(14) ωc1=ωc11,ωc12,ωc13=0.2557,0.5584,0.1859,ωc2=ωc21,ωc22,ωc23,ωc24=0.2162,0.1674,0.3344,0.2820,ωc5=ωc51,ωc52,ωc53=0.3554,0.2206,0.4240.

*(6) Determination of Risk Level.* Calculate the comprehensive correlation matrix *K*_1_ of sample 1:

(15) $$\begin{array}{c} K_{1}=\left[\begin{array}{c} \omega_{B_{1}}\cdot K_{11}\\ \omega_{B_{2}}\cdot K_{12}\\ \omega_{B_{3}}\cdot K_{13}\\ \begin{array}{c} \omega_{B_{4}}\cdot K_{14}\\ \omega_{B_{5}}\cdot K_{15} \end{array} \end{array}\right]=\left[\begin{array}{ccc} 0.1167 & -0.2115 & -0.4823\\ 0.2351 & -0.3918 & -0.5272\\ -0.1983 & 0.2333 & -0.0700\\ \begin{array}{c} -0.4286\\ 0.2168 \end{array} & \begin{array}{c} -0.5714\\ -0.2796 \end{array} & \begin{array}{c} -0.6250\\ -0.5291 \end{array} \end{array}\right], \end{array}$$

while the criterion layer weights given by experts are

(16) ωB=ωB1,ωB2,ωB3,ωB4,ωB5=0.2360,0.2102,0.2404,0.1851,0.1283.

Using formula (11) to calculate the comprehensive correlative degree *K*_1_ of sample 1, the value is (−0.0222, −0.2179, −0.4250). It can be known that sample 1 is at a low risk level. In the same way calculate the comprehensive correlative degree of sample 2, sample 3, and sample 4 as *K*_2_ = (0.0208,  −0.0905,  −0.6011), *K*_3_ = (0.1516,  0.0373,   − 0.2570), and *K*_4_ = (−0.1053,  0.1534,  −0.0918), respectively. The results of risk evaluation of sample 2, sample 3, and sample 4 are low risk, low risk, and medium risk, respectively. This is in high conformity with expert judgment.

## 5. Conclusion

Bogie system is a key system in the safe operation of a train. Accurate risk assessment can effectively prevent the occurrence of accidents, reduce part damage, and increase the utility of trains. This paper presents a risk assessment method of the bogie system based on extension theory and the entropy weight method. We consider the function and importance of various components of the bogie system and establish a risk evaluation method for the bogie system. Risk assessment results of different sample data of bogie system show that this method has good accuracy and can effectively determine the risk status of the bogie system.

The method carries out evaluation for each index and can not only evaluate a single index but also evaluate multiple indices. But determination of the standard domain and extensional domain is needed during evaluation. At present there is no clear and effective method to define the threshold value. It is defined mainly by sample data training and commonly used work standard. Scientific and effective determination of grade threshold is one of the subsequent works that is needed. In addition, the revision and perfection of the index system of elements are needed in follow-up research.

## Figures and Tables

**Table 1:** Index standards.

Evaluation index	Low risk	Medium risk	High risk
Effective value	[0.05, 0.85]	(0.085, 0.1]	(0.1, 1)
Peak factor	[2.5, 3.2]	(3.2, 3.5]	(3.5, 30)
Kurtosis	[2.5, 3.5]	(3.5, 4]	(4, 30)
Wheel diameter	(780, 840]	(775, 780]	[770, 775]
Wheel rim width	(29, 32]	(28, 29]	[26, 28]
Difference of coaxial wheel diameter	[0, 1.5)	[1.5, 2)	[2, 10)
Difference of co-bogie wheel diameter	[0, 3)	[3, 4)	[4, 15)
Traction motor temperature	[0, 70)	[70, 100)	[100, 300)
Brake shoe thickness	[20, 55]	[15, 20)	[0, 15)
Risk of elastic suspension device	[0, 50)	[50, 75)	[75, 100)
Risk of bogie frame	[0, 50)	[50, 75)	[75, 100)
Risk of ground device	[0, 50)	[50, 75)	[75, 100)

**Table 2:** Four groups of sample data.

Evaluation index	Sample 1	Sample 2	Sample 3	Sample 4
Effective value	0.0644	0.0741	0.0917	0.0566
Peak factor	3.2241	3.3622	3.5621	3.3541
Kurtosis	2.7301	2.9901	2.7782	3.0803
Wheel diameter	791.5	786.2	782.3	782.9
Wheel rim width	31.6	29.1	27.2	27.3
Difference of coaxial wheel diameter	0.9	1.8	0.8	1.7
Difference of co-bogie wheel diameter	2.6	3.9	3.85	1.7
Traction motor temperature	93	61.6	92.8	52
Brake shoe thickness	40	35	17	19
Risk of elastic suspension device	30	24	26	23
Risk of bogie frame	15	18	29	20
Risk of ground device	51	40	55	47
